# Psychiatric comorbidities in asperger syndrome and high functioning autism: diagnostic challenges

**DOI:** 10.1186/1744-859X-11-16

**Published:** 2012-06-25

**Authors:** Luigi Mazzone, Liliana Ruta, Laura Reale

**Affiliations:** 1 Child Neuropsychiatry Unit, Department of Neuroscience, I.R.C.C.S. Children’s Hospital, Bambino Gesù, Rome, Italy; 2 Autism Research Centre, Department of Psychiatry, Cambridge University, Douglas House,18B Trumpington Rd, Cambridge CB2 8AH, UK; 3 Division of Child Neurology and Psychiatry, Department of Pediatrics, University of Catania, Catania, Italy

**Keywords:** Asperger, High functioning autism, Comorbidities, Diagnostic challenges

## Abstract

Several psychiatric conditions, both internalizing and externalizing, have been documented in comorbidity with Asperger Syndrome (AS) and High Functioning Autism (HFA). In this review we examine the interplay between psychiatric comorbidities and AS/HFA. In particular, we will focus our attention on three main issues. First, we examine which psychiatric disorders are more frequently associated with AS/HFA. Second, we review which diagnostic tools are currently available for clinicians to investigate and diagnose the associated psychiatric disorders in individuals with AS/HFA. Third, we discuss the challenges that clinicians and researchers face in trying to determine whether the psychiatric symptoms are phenotypic manifestations of AS/HFA or rather they are the expression of a distinct, though comorbid, disorder. We will also consider the role played by the environment in the manifestation and interpretation of these symptoms. Finally, we will propose some strategies to try to address these issues, and we will discuss therapeutic implications.

## Introduction

Asperger Syndrome (AS) and High Functioning Autism (HFA) are two conditions within the broad category of the Autism Spectrum Disorders (ASDs) that are often overlapping and characterized by social-communication impairment and over-focused, repetitive interests and behaviours, without any significant learning disabilities or language delay in the case of AS. Individuals suffering from AS/HFA typically show pedantic speech often with monotonous or exaggerated vocal intonation [1], poor nonverbal communication [2] and motor clumsiness. Despite AS and classic autism both belonging to the same category of ASDs, individuals with AS tend to show a distinct pattern of social impairment that seems to be milder than in classic autism [3], and it has been hypothesized that the differences between AS and classic autism may be both quantitative and qualitative [4].

The management of behavioral problems in children and adolescents with autism spectrum disorders is a challenge for clinicians and families and the psychiatric symptoms in comorbidities could even exacerbate the behavioral dyscontrol [5-7]. Individuals with AS and HFA may show an impairment in describing their own feelings and emotions [4], so it is not easy to detect and recognize another psychiatric comorbidity that could be masked by the autistic symptoms themselves. One of the main problems with individuals suffering from AS/HFA is that behavioral symptoms due to one of the comorbid conditions that often run together with this type of ASD (see section “AS/HFA and comorbid psychiatric conditions” and Table 1) could arise in different social environments, including family and school, and during social activities. For these and other reasons in the daily clinical practice it is difficult to make a decision about the most appropriate diagnosis and therapeutic strategies.

**Table 1:** Summary of studies published between 2000–2011 exploring psychiatric comorbidity in asperger syndrome and high functioning autism

	**Study**	**Type of study**	**Sample characteristic**^**a**^** N/Age (M ± SD)**	**Control Group(s)b N/Age (M ± SD)**	**Assessment of psychiatric comorbidityc**	**Findings**
DEPRESSION	Green et al., 2000 [6]	Comparison between AS and CD.	20 AS (13.75) Range: 11-19	20 CD (14.47) Range: 11-19	ICD-10	Asperger showed higher rates of depression than controls. Chronic unhappiness and loneliness were more commonly associated with AS.
	Kim et al., 2000 [7]	Follow-up to clinic series.	19 AS 40 HFARange: 9-14	1751 CG Range: 9-14	OCHS-R	17% had clinically relevant depression scores. Depression correlated with anxiousness and externalizing behavior.
	Barnhill, 2001 [8]	Descriptive study of depressive symptoms.	33 AS Range: 12-17	None	CDI	54% reported depressive symptoms, with a significant positive relationship between depression symptoms and ability attribution for social failure.
	Hedley et al., 2006 [9]	Relationship between social comparison processes and depressive symptoms.	36 AS (12.9 ± 1.94) Range: 10-16	None	SCS CDI	Participants who perceived themselves as being more dissimal to others reported higher depressive symptoms.
	Meyer et al., 2006 [10]	Cross-selectional study about depression in AS.	31 AS (10.1 ± 1.9)	33 CG (10.2 ± 1.9)	BASC-SRP BASC-PRS	The children themselves reported greater symptoms of anxiety and depression.
	Whitehouse et al., 2009 [11]	Relationship between loneliness and depressive symptoms in AS	35 AS (14.2 ± 0.8)	35 CG (14.4 ± 0.10)	CES-DC	Around two thirds (n = 23) of the adolescent with AS self-reported significantly higher levels of depressive symptoms than control group.
	Lugnegård et al., 2011 [12]	Evaluation of psychiatric comorbidity	54 AS (27.0 ± 3.9)	None	SCID	70% had experienced at least one episode of major depression, and 27 of these (50% of the total group) had recurrent major depressions.
BIPOLAR	Munesue et al., 2008 [13]	Study of mood disorders in consecutive outpatients.	44 HFA Range: 13-39	None	DSM-IV	Sixteen patients (36.4%) were diagnosed with mood disorder. Among these bipolar disorder accounted for 75% of cases.
	Hofvander et al., 2009 [14]	Evaluation of psychiatric and psychosocial problems in ASD with normal IQ	5AD 67 AS 50 PDD-NOS Range: 16-60	None	DSM-IV	In the AS subgroup the most common life-time comorbid condition was mood disorder (n = 65, 53%). Criteria for a bipolar disorder (BP) were met by 10 subjects (8%), five of whom had bipolar I subtype and two bipolar II, while three were coded as unknown subtypes.
	Lugnegård et al., 2011 [12]	Evaluation of psychiatric comorbidity	54 AS (27.0 ± 3.9)	None	SCID	Five participants (9% of total group) met criteria for bipolar II disorder, whereas none met criteria for bipolar I disorder.
ANXIETY	Green et al., 2000 [6]	Comparison between AS and CD.	20 AS(13.75) Range: 11-19	20 CD (14.47) Range: 11-19	ICD-10	Asperger showed more symptoms of general anxiety and OCD than CD
	Gillott et al., 2001 [15]	Measures of anxiety and social worries.	15 HFA Range: 8-12	30 CG Range: 8-12	SCAS SWQ	HFA group showed higher rates of anxiety than in typically developing or language impaired children. 7 of 15 children with HFA were at or above clinical mean score.
	Burnette et al., 2005 [16]	Relations between the weak central coherence hypothesis, theory of mind skills, and social-emotional functioning.	23 HFA (11. 25 ± 1.57)	20 CG (11.00 ± 1.32)	BASC-SRP SASC-R	Autistic subjects self-reported significantly more social anxiety symptoms
	Pfeiffer et al., 2005 [17]	Relationships between sensory modulation and symptoms of affective disorders.	50 AS Range: 6-17	None	MASC	Significant correlation was found between sensory defensiveness and anxiety in children with AS
	Russell and Sofronoff, 2005 [18]	Examination of anxiety and social worries in AS.	65 AS Range: 10-13	261 CG Range: 10-13	SCAS SWQ	AS self-reported levels of anxiety symptoms significantly higher than control group
	Meyer et al., 2006 [10]	Cross-selectional study about anxiety in AS.	31 AS (10.1 ± 1.9)	33 CG (10.2 ± 1.9)	BASC-SRP BASC-PRS	AS self-reported high social anxiety levels correlating with their social perception, understanding and experience.
	Sukhodolsky et al., 2008 [19]	Study of the frequency of anxiety symptoms in PDD.	171 ASD (8.2 ± 2.6)	None	CASI	73 (43%) had a pathological scores for at least one anxiety disorder. Higher levels of anxiety were associated with higher IQ, functional language use, and stereotyped behaviors.
	Kuusikko et al., 2008 [20]	Association between AS/HFA and social anxiety symptoms.	54 HFA/AS (11.2 ± 2.2)	305 CG (12.2 ± 2.2)	SPAI-C SASC-R CBCL	Adolescents with HFA/AS suffered from more symptoms of social anxiety than the community sample on all measures.
	Lugnegård et al., 2011 [12]	Evaluation of psychiatric comorbidity	54 AS (27.0 ± 3.9)	None	SCID	56% met criteria for at least one anxiety disorder, and 11 of these fulfilled diagnostic criteria for two or more anxiety disorder diagnoses. 22% had social anxiety disorder, 22% had generalized anxiety disorder, 13% had panic disorder, 15% had agoraphobia and 7% had OCD.
OCD	Russell et al., 2005 [21]	Study of Obsessive Repetitive behaviours in AS and OCD groups.	40 AS/HFA (27.9 ± 8.5)	45 OCD (36.6 ± 11.5)	Y-BOCS	The two groups had similar frequencies of obsessive-compulsive symptoms. Somatic obsession and repeating rituals were more common in the OCD group.
	South et al., 2005 [22]	Study of the four repetitive behavior categories, comparing AS/HFA and CG.	19 AS (14.10±3.47), 21 HFA (14.28±3.02)	21 CG (13.34 ± 3.28)	RBI, YSII	The CG showed lower scores than AS/HFA for all four repetitive behavior categories: Object Use; Motor Movements; Rigid Routines and Circumscribed Interests.
	Zandt et al., 2007 [23]	Study of the types of ripetitive behaviours in ASD.	19 ASD (10.97 ± 2.42)	17 OCD (12.30 ± 2.17), 18 TD (11.942.94)	RBQ, CY-BOCS	Children with ASD reported more compulsions and obsessions than typically developing children. Consideration of the type of compulsions and obsessions in each disorder suggests that the compulsions in ASD tended to be less sophisticated.
	Ruta et al., 2009 [24]	Examination of the occurrence and characteristic features of obsessive–compulsive behaviours.	18 AS (10.61 ± 1.91)	20 OCD (11.65 ± 1.69), 22 TD (10.68 ± 2.01)	CY-BOCS	The AS group displayed slightly higher frequencies of Saving/Hoarding and Ordering clusters compared to OCD group.
	Mack et al., 2010 [25]	Clinical characteristics and symptoms severity in patients with OCD + AS/HFA; OCD + TS or OCD alone.	12AS/HFA + OCD Range:12-18	12 OCD + TS Range: 9–17, 12 OCD Range: 9-17	Y-BOCS	Children with ASD may experience a similar level of impairment, equally distressing, time consuming and contributing to a similar level of interference in functioning from obsessive/compulsive symptoms as compared to children with TS plus OCD and children with OCD only.
	Lai et al., 2011 [26]	Behavioral Comparison of Male and Female Adults with High Functioning Autism Disorders.	68 AS/HFA Range: 18-45	None	BAI, BDI, OCI-R	A significant proportion of adults with AS/HFA showed clinically significant anxiety, depression, or obsessive-compulsive symptoms. Overall male and female adults with ASC were not different on these symptoms.
ADHD	Holtman et al., 2005 [27]	Study of the frequency of ADHD-like symptoms in AS/HFA	104 AS/HFA Range: 3.4-20.2	None	CBCL	Sixty-five percent of the subjects scored above the clinical cut-off on the attention problems scale
	Tani et al., 2006 [28]	Exploration of childhood ADHD problems retrospectively	20 AS (27.2 ± 7.3)	10 TD (26.5 ± 8.1)	WURS	AS group displayed higher scores as indicated from self- and parents-report.
	Simonoff et al., 2008 [29]	Assessment of psychiatric disorders in ASD	112 ASD (11.5) Range: 10–13.9	None	DSM-IV	70.8% of ASD children had at least one current psychiatric disorder. The most common comorbidities were social anxiety (29.2%), ADHD (28.1%), and oppositional defiant disorder (28.1%).
	Mattila et al., 2010 [30]	Community- and clinic-based study exploring comorbid psychiatry in AS/HFA	50 AS/HFA (12.7 ± 1.5)	None	K-SADS-PL CGAS	One or more comorbid psychiatric disorders were diagnosed in 74% of the cases. The most common disorders were behavioural (44%), anxiety (42%) and tic disorders (26%).
	Mukaddes et al., 2010 [31]	Comparison the rate and type of psychiatric comorbidity in children and adolescents HFA/AS	30 AS, 30 HFA	None	K-SADS-PL	The rate of comorbid psychiatric disorders was very high in both groups. The most common disorder in both groups was attention deficit hyperactivity disorder. The AS group displayed greater comorbidity with depressive disorders and ADHD.

^a^ Sample characteristic: AS = Asperger’s Syndrome, HFA = High-functioning Autism, ASD = Autism Spectrum Disorders, PDD-NOS = Pervasive Developmental Disorder-Not otherwise Specified.

^b^ Control Group(s): *CD* = Conduct Disorder, *AD* = Autistic Disorder, *CG* = Control Group, *OCD* = obsessive Compulsive Disorder, *TD* = Typically developing, *TS* = Tourette Syndrome.

^c^ Assessment of psychiatric comorbidity: *ICD*-10 = Tenth revision of the International classification of disease, *OCHS-R* = Ontario Child Health Study-Revised, *CDI* = Children’s Depression Inventory, *SCS* = Social Comparison Scale, *BASC-SRP* = Behaviour Assessment System for Children-Self Report of Personality, *BASC-PRS* = Behaviour Assessment System for Children-Parent Report Scale, *CES-DC* = Centre for Epidemiological Studies Depression Scale, *SCID* = Structured Clinical Interview for DSM-IV Axis I Disorders, *DSM-IV* = Diagnostic and statstical manual of mental disorders (4th ed.), *SCAS* = Spence Children's Anxiety Scale, *SWQ* = Spence Social Worries Questionnaire, *SASC-R* = Social Anxiety Scale for Children-Revised, *MASC* = Multidimensional Anxiety Scale for Children, *CASI* = Child and Adolescent Symptom Inventory, *SPAI-C* = Social Phobia and Anxiety Inventory for Children, *CBCL* = Child Behavior Checklist, Y-*BOCS* = Yale Brown Obsessive Compulsive Scale and Symptom Checklist, *RBI* = Repetitive Behavior Interview, *YSII* = Yale Special Interests Interview, *RBQ* = Repetitive Behaviour Questionnaire, *CY-BOCS* = Children's Yale Brown Obsessive-Compulsive Scale, *BAI* = Beck Anxiety Inventory, *BDI* = Beck Depression Inventory, *OCI-R* = Obsessive Compulsive Inventory-Revised, *WURS* = Wender-Utah Rating Scale, *K-SADS-PL* = Schedule for Affective Disorders and Schizophrenia for School-Age Children, Present and Lifetime Version, *CGAS* = Children’s Global Assessment Scale.

In this review we examine the interplay between common psychiatric comorbidities and AS/HFA. In particular, we will discuss which psychiatric disorders have been more frequently reported in association with AS/HFA. We will also point out the difficulties that clinicians and researchers have to face when trying to make the correct diagnosis of a comorbid condition in AS/HFA basing on the currently available psychometric tools (i.e. scales, checklists and questionnaire).

Furthermore, we will discuss the important role played by the environment and finally we will outline some useful strategies to address these issues and challenges for therapeutic implications.

## Methods

A systematic literature search was performed using PubMed to identify clinical studies that assess psychiatric comorbidities in individuals with AS or HFA. Trial quality was assessed according to a standardized and validated set of criteria.

In details, we searched Medline databases from January, 2000, to December, 2011, with the search terms “autism”, “autistic disorder”, “pervasive developmental disorder”, “autism spectrum disorder”, and “Asperger syndrome” in combination with “comorbidity”, “depression”, “anxiety”, “bipolar disorder”, “adolescent”, “childhood”, “obsessive compulsive disorder”, “attention deficit hyperactivity disorder”, and “conduct disorder”. We also searched references from recent reviews and other reports identified by this search strategy, and selected those we judged relevant. Review articles and book chapters are cited to provide more details and references.

### Study selection

We selected only studies that concerned psychiatric comorbidity in individuals with AS or HFA, according to the following criteria: 1) only clinical studies were included; 2) the diagnosis of AS or HFA had to be made with DSM or ICD criteria; 3) the use of specific tools for the diagnosis, as ADI and ADOS tests was not required, but AS/HFA diagnoses had to be described in the methods section of the study; 4) a separate analysis describing the eventual psychiatric comorbidity of all subjects included in the study in a standardized, transparent, and reliable manner was also required.

Two authors (Drs Mazzone and Reale) independently screened for eligibility each of the studies identified by the literature search. All potentially relevant studies were retrieved as full articles and then again independently reviewed by these two authors.

### Study-quality assessment

The quality of each study was assessed according to a standardized and validated set of criteria based on the protocols of the Cochrane Database of Systematic Reviews as used in randomized, controlled trials [32] and modified to cover the case–control design of the studies included in this review: 1) comparison group(s): the presence of at least 1 comparison group, preferably a sample of healthy children from the same region as the children included in the study; 2) sample size: on the basis of power analysis a sample size of >10 participants per comparison group was required; 3) sample selection: a random selection strategy had to be used; 4) design: the investigation had to be case-controlled and based on quantitative information; 5) comorbidity measures: these measures had to be standardized, reliable, and valid and cover the eventual psychiatric comorbidity; 6) statistical analyses: hypothesis testing using appropriate statistical analyses had to be performed.

These 6 criteria were assessed and scored independently by two investigators (Drs Mazzone and Reale). A score of 1 (criterion met) or 0 (criterion not met) was used, which led to a total maximum score of 6 points per study. This process led to a uniform score on all included articles. A score of 4 or above was considered “relevant”.

## AS/HFA and comorbid psychiatric conditions

### Internalizing disorders

As illustrated in Table 1, several studies reported an association between AS/HFA and internalizing symptoms, in particular depression [6-11], bipolar disorders [13] and anxiety [33,34]. Interestingly, a bidirectional association has been detected between internalizing disorders and autistic symptoms: both an higher prevalence of anxiety disorders has been found in AS [10,35] and a higher rate of autistic traits has been reported in youths with mood and anxiety disorders [36]. Another study showed that people with AS displayed more social anxiety symptoms compared to healthy control individuals, even if these symptoms were clinically overlapping with the characteristic social problems, typical of AS [20].

Another documented association is with Obsessive-Compulsive Disorder (OCD), although it is difficult to discriminate whether the obsessive-repetitive behaviors are more likely to be the expression of a separate, comorbid OCD, or rather should be addressed as an integral part of the core symptoms of AS [21]. In a previous study, we examined the presence of obsessive–compulsive symptoms in children with AS compared with typically developing children and children with obsessive-compulsive disorders (OCD). We found in the AS group significantly higher frequencies of compulsive hoarding, repeating and ordering compared to controls, while the OCD group reported significantly higher frequencies of contamination and aggressive obsessions and checking compulsions compared to both the AS group and the control group. Finally, in our sample, neither the OCD group nor the AS group demonstrated a completely full awareness of the intrusive, unreasonable and distressing nature of symptoms and the level of insight did not differ between the OCD group and control group, although an absence of insight was observed in the AS group [24]. Also others studies have systematically compared repetitive behaviors in children with ASD and OCD. In their study, Russell et al., found a similar pattern of obsessions and compulsions in the HFA/AS and OCD groups, with 25% of HFA/AS individuals receiving a formal diagnosis of OCD according to ICD-10 criteria [21]. Another study showed that OCD reported more frequent and sophisticated obsessions and compulsions than the HFA and that both groups showed more obsessions and compulsions than a typically developing comparison group [23].

Indirect evidence for an association between ASDs and OCD comes from the studies of serotonin dysfunction in psychiatric disorders. Various studies have suggested that serotonergic abnormalities occur in both ASDs and OCD. In addition, medications that are effective in the treatment of OCD in the general population have been found to be useful in some patients with ASDs, especially in the control of ritualistic behaviors [37]. The association between ASDs and OCD as well as other psychiatric symptoms including anxiety seems to be further supported by the observations that some brain regions such as the amygdala play a crucial role in ASDs, in relation to abnormal fears, compulsive behaviors and increased anxiety [38,39].

### Externalizing disorders

An association between AS/HFA and attention deficit hyperactivity disorder (ADHD) and other externalizing disorders such as disruptive behavior and conduct disorders [28,36,40-42] have been reported. According to DSM-IV criteria, it is not possible to perform the diagnosis of ADHD in the context of an ASD [43]. However, the debate about ADHD comorbidity in ASDs is still open in the DSM-V proposal and clinical opinion, research practice and theoretical models seem to suggest that comorbidity between these disorders is a real, relevant, and frequent occurrence [44]. Furthermore a phenotypic overlap between the AS/HFA and ADHD has been recently discussed and the controversy whether these two conditions take part of a spectrum of symptoms is still also open [27,42]. Even though an option being considered on DSM-V regarding exclusion criteria for ADHD may be to remove ASD from the exclusion criteria, whether DSM-V will allow a comorbid ASD/ADHD diagnosis remains uncertain. Indeed, there is no evidence that ADHD is inconsistent with ASDs and when an association between ADHD and these disorders exists, it responds to treatment similar to ADHD without ASD. In the clinical practice it is often possible to recognize ADHD-like symptoms such as inattentive symptoms, in children with AS/HFA [45,46] and neuropsychological studies have reported that children with AS/HFA show the same inattention pattern of children with ADHD in terms of type and degree [47,48]. However, other studies showed that inattention symptoms in school settings among children with ASD were more likely to be attributed to low IQ [49].

Another controversial association in the context of externalizing symptoms regards the relationship between AS/HFA and psychopathy, that has been strongly suggested by some studies showing an increased risk for crimes in AS, that may be attributable to either a lack of insight typical of AS, and/or to co-occurring psychiatric disorders [6,41,50], and further supported by the fact that most of the individuals suffering from AS who commit violent crime also show additional psychiatric disorders [41].

Indeed, recent findings suggest that high-functioning autism disorders are over-represented in the criminal population as compared to the general population [51] and that the unique features of the autism functioning may heighten their risks for engaging in criminal behavior.

However, this association does not seem to be so clear, and contrasting results have been found in different studies looking at the incidence of antisocial behaviors in individuals with AS/HFA For instance, based on data from the original studies by Hans Asperger, individuals with AS/HFA are not more likely to became offender or behave antisocially than any other group in the general population. In accordance with this observation, another study failed to detect higher rates of criminal behavior in AS [52], thus making this association highly controversial. Both individuals with AS/HFA and those with antisocial conduct disorder can have deficit in empathy towards others, which may contribute to antisocial behavior [6,53]. In fact, people presenting antisocial behaviors show reduced impulse control, deficit of emotional empathy and little, and few signs of guilt or remorse [41]. However, antisocial, non-ASD individuals retain the capacity of understanding the minds of others, and can use this knowledge for manipulation and control. By contrast, a key feature of ASD is the impairment in developing an age appropriate “theory of the mind”. For these reasons, the mechanisms underlying antisocial acts are quite different in AS compared to antisocial individuals [53]. Indeed, their deficit in having a "theory of mind" impairs their social judgment and increases the risk of violating norms and laws. On the other hand, individuals with AS often show a strong sense of right and wrong, and once they have understood the rules they are likely to stick to them more rigidly than most people [54].

### Tic disorders and other disorders

Tourette Syndrome (TS) and other tic disorders have been found in comorbidity with AS [55,56]. A Swedish study showed that 20% of all school-age children with AS met full criteria for TS [57]. However, some authors consider this comorbidity as a predictor of a better outcome for autistic symptoms, even if the data collected to date are not sufficient to confirm this hypothesis [58]. Despite autism has been considered for many years a sort of early psychosis there are not many studies investigating the association between psychotic symptoms and autism. Ghazziudin et al. (1995), studying how many children with ASD referred for psychotic behavior are given a diagnosis of schizophrenia, showed that when psychotic behaviors were the presenting symptoms in autistic children (especially those who were higher-functioning) depression, and not schizophrenia, was the likely diagnosis [59]. Thus, it’s likely that individuals with high-functioning autism and Asperger syndrome have characteristics that could lead to a mistaken diagnosis of schizophrenia and other psychotic disorders. However, there are some findings showing theory of mind deficits [60,61] and mirror neuron [62] impairment in both disorders that could suggest a certain degree of overlap. Furthermore similar connectivity deficits have been reported in functional imaging studies, and both disorders share several genetic signals that are being identified through the detection of copy number variants [63].

Finally people with AS/HFA, in clinical practice, could meet criteria also for a personality disorder such as paranoid, schizoid [64] and schizotypical personality disorders [65]. However these conditions are usually first diagnosed in adolescence or adult life and cannot be diagnosed in the context of an ASD disorders.

## Difficulties in performing a diagnosis of psychiatric comorbidity in individuals with AS/HFA

Recognizing psychiatric comorbidities in individuals with AS/HFA can be challenging for clinicians. Considering that individuals with AS/HFA may show an impairment in processing and describing their own feelings and emotions, the clinical information is often obtained by interviewing family members rather than the AS participants themselves or detected from a direct observation in their social environment.

Moreover, the symptoms of psychopathological conditions may be masked by those typical of AS/HFA and the threshold between autism spectrum core symptoms and comorbid symptoms can be blurred. For instance, a sudden decrease of repetitive and obsessive behaviors in individuals with AS may represent a manifestation of depressive symptoms, but could also be mistakenly ascribed to an improvement in one of the diagnostic dimensions of AS itself [66]. In the clinical practice, various psychometric instruments, such as clinical interviews, self-report questionnaires and checklists, are widely used to assist in the diagnosis and they constitute a valuable support for clinicians. However, these diagnostic tools have been designed and standardized to spot different clusters of psychopathological symptoms referring to the general population and they may not be appropriate to AS/HFA.

For this reason, not only the validity of these instruments need to be tested in ASDs, but also their administration may be problematic in individuals with AS/HFA because of their difficulties to sustain a reciprocal conversation, to talk about their self, to report events and their lack of understanding and empathy for the feelings of other people. All these issues render the correct interpretation of the answers even more complicated, thus misleading the real nature of symptoms in comorbidity.

There are currently no scales specifically designed to evaluate psychiatric comorbidity in persons with ASDs, and the studies that investigated comorbid disorders in AS/HFA either referred to the DSM-IV diagnostic criteria, or tried to adapt the scales used for the general population, and this obviously has several limitations. In one of these studies, Hedley & Young investigated the link between social comparison processes and depression in children and adolescents with AS [9], by using the Children's Depression Inventory (CDI) to detect depressive symptoms. The CDI is a self- report scale, which investigates the presence of negative moods, interpersonal difficulties, negative self-esteem, and ineffectiveness and implies intact abilities to understand and report about internal state of mind, feeling and emotions. These abilities can be, to various degrees, impaired in AS, thus decreasing the sensitivity of the clinical assessment. For instance, items 11, 12 and 25 refer to the sense of self in the social interactions, and may be altered per se in AS/HFA individuals without being a sign of depression. The same might account for items 20, 21 and 22, which presuppose children's comprehension of different perceptual perspectives and preserved mental ability to infer mental states from people’s behavior, another capability that might be impaired per se in AS. Another study investigated the presence of DSM-IV-defined bipolar disorder in adolescents and young adults with HFA [13]. According to this study it seems that adolescents and young adults with HFA show a higher prevalence of bipolar disorders as compared to controls. Most of the studies addressing the presence of psychiatric conditions in ASDs are made by listing, with a categorical approach, the presence of certain target symptoms, without extensively describing the type and peculiarity of those symptoms in the specificity of HFA neuropsychological profile. This constitutes a critical issue and raises the question of whether the diagnostic criteria for psychiatric conditions listed in the DSM IV-TR or ICD-10 can be applied to ASDs or need to be revised and better contextualized. For example, signs of manic and hypomanic mood are the core symptoms of bipolar disorder according to DSM-IV. In the context of ASDs, however, it is difficult to define manic or hypomanic mood. Although, according to Leibenluft and colleagues (2003) criteria for the broad phenotype of juvenile mania, symptoms such as irritable or anger mood, excessive reactivity and hyperarousal (agitation, racing thoughts/flight of ideas, pressured speech) could be strongly suggestive of a comorbid severe mood dysregulation disorder [67,68], on the other hand, the impairment to modulate the arousal state, especially in social situations, may lead to motor overexcitement, laugh bursts or exacerbation of body stereotypies and mannerisms that may simply well depend on impairments in social understanding.

Anxiety symptoms have also been documented in individuals with AS/HFA. An interesting study performed on adolescents with AS/HFA showed an age-related increase in social anxiety within the HFA/AS group; by contrast, a decrease of behavioral avoidance by age was seen in control participants [69]. In this study, the Social Phobia and Anxiety Inventory for Children (SPAI-C), the Social Anxiety Scale for Children - Revised (SASC-R), and the Child Behavior Checklist (CBCL) were administered to participants. The Child Behavior Checklist (CBCL) is indeed an important scale to perform psychopathological assessment of psychiatric comorbidities. However, the validity of this scale in AS/HFA is controversial with some studies considering some items of the CBCL (i.e., repeats acts, strange behavior, strange ideas, and withdrawn) extremely useful in identifying children with ASDs [69], whereas others documenting reliability in only a subgroup of individuals with ASDs [70]. Once again, these studies arise the clear need for psychometric tools designed specifically to assess anxiety disorders in the ASD population, keeping in mind that participants with AS/HFA may have a compromised sociability, that could provide a certain level of social anxiety, without necessarily meaning the presence of an overlapping anxiety disorder. On the other hand, a compromised sociability would not necessarily lead to anxiety, especially in individuals with AS/HFA that do not socialize in the same way and may not become anxious. In this context, anxiety may be a separate dimension.

One of the most controversial comorbidities, in children with AS/HFA, is the co-occurrence of Attention Deficit Hyperactivity Disorder (ADHD), and this is mainly due to specific diagnostic difficulties in the clinical practices. However according to the DSM-IV co-diagnosis of AS and ADHD cannot be done, thus further complicating the diagnostic process. Indeed, study results from the literature on this issue are controversial, because of the lack of validated scales to investigate ADHD symptoms in AS/HFA. For instance, one of these studies showed an higher prevalence of ADHD symptoms - investigated retrospectively by a self-report scale - in AS participants, [28]; however, the risk of recall bias or biased self-awareness about ADHD symptoms in AS/HFA group may represent a threat to the internal validity of this kind of investigations.

Another important issue regarding the comorbidity in AS/HFA is represented by the broader spectrum of Obsessive Compulsive and related disorders [71]. Symptom overlapping among OCD and AS/HFA makes it difficult to clearly differentiate the clinical phenomenology of obsessive- compulsive and repetitive behaviors in AS/HFA. The development of appropriate scales adapted for ASDs are warranted to shed light on the various aspects of this comorbidity. An instrument that could help to perform diagnosis of OCD comorbidity in ASDs is the Children's Yale-Brown Obsessive Compulsive Scales (CYBOCS), modified for pervasive developmental disorders [72], validated by raters from five Research Units on Pediatric Psychopharmacology (RUPP) Autism Network that were specifically trained to reliability. However, this scale has been developed in particular for low functioning participants and not for AS/HFA individuals.

In our point of view, the CYBOCS modified for ASDs represents a good example of a reliable specific measure to be used to assist the diagnosis of OCD comorbidity in ASDs. It also indicates that already existing tools may be successfully modified to be applied to AS/HFA individuals in a reliable way.

## Psychiatric symptoms are phenotypic manifestation of AS/HFA, or are they rather due to the expression of a separate and comorbid disorder?

Another common challenge for clinicians is to determine if psychiatric symptoms observed in AS/HFA are part of the same dimension – i.e. the Autism Spectrum itself – or rather represent different categorical factors – i.e. a psychiatric disorder in comorbidity. This happens because, as mentioned above, the core symptoms of ASDs often mask the symptoms of the comorbid condition. A helpful strategy to disentangle this issue could be to look prospectively at the overall clinical outcome through longitudinal studies.

Longitudinal studies allow to closely follow the developmental trajectories of AS\HFA and to detect subtle changes in behavior at different stages of development in terms of progressive appearance of clinical symptoms and impact of life experiences.

Unfortunately, most of the clinical studies on AS/HFA conducted so far typically have been cross-sectional, comparing one particular clinical measure at a single time point across samples. The interpretation of these cross-sectional findings is mostly focused on the abnormalities in the patient group as compared to healthy controls and on the contributions that these abnormal clinical abnormalities may have on disease evolution. If the need for longitudinal, prospective research is common to the study of all developmental psychopathology, it is especially crucial for AS/HFA in order to understand the lifetime history of the disorder and the role of comorbid disorders. Indeed, children suffering from AS have a different pattern of development compared to normal children and their life experiences influence their cognitive and emotional status beginning within their first years of life. For instance, numerous cross-sectional studies that investigated the presence of mood and anxiety disorders in AS, found that the rate of depression among children with Asperger syndrome is higher than within a community control group [7]. Anxiety and depression are highly related, and high levels of anxiety have been shown to predispose to depression [73,74]. However cross-sectional studies do not provide any kind of information about different degrees of remitting versus non-remitting symptoms, as well as about the different etiological subtypes of depression.

Therefore, there is a clear need for longitudinal population studies to identify whether individuals with high anxiety levels and AS/HFA have increased susceptibility for depression during lifetime.

The correct identification and attribution of depressive symptoms in individuals with AS/HFA as well as their change over time is crucial to avoid inaccurate diagnostic interpretation. For instance, it is well documented [75] that the onset of depression in individuals with AS is usually, if not always, associated with a decrease in repetitive and obsessive behaviors, a reduction that may be considered as an improvement of an OCD, rather than a co-occurring depressive condition. In this context, longitudinal studies could be helpful to monitor and understand the lifetime course of psychiatric symptoms in these patients, thus allowing a better discrimination between different comorbid situations. Longitudinal studies could also be useful to better clarify the association with ADHD, that, if properly investigated, can be frequently found in comorbidity with AS. Various neuropsychological studies reported that individuals with AS show different patterns of manifestation of ADHD symptoms [76]. However, the developmental trajectory of attention problems and motor hyperactivity symptoms development in relation to age, is still not completely clear, even if some studies suggest that certain symptoms are more likely associated with specific periods of life (i.e. preschool years or adolescence) [5]. Longitudinal studies that would follow these individuals across time could help to better clarify the dynamics of this comorbidity in AS/HFA.

Hence, in our opinion, although cross-sectional studies are extremely useful for generating hypotheses, these hypotheses in turn need to be further confirmed by longitudinal investigations. The development of lifespan approaches could help to clarify the core of pathophysiological processes of the main disorder in ASDs, thus allowing an easier discrimination between those symptoms that are part of the AS/HFA clinical phenotype from those that are the expression of a comorbid disorder.

## The role of the environment in the manifestation of psychiatric disorders in AS/HFA

The environmental context differentially affects individuals with AS/HFA and it is an additional crucial player that may influence the onset, expression and severity of the psychiatric disorder in comorbidity. Indeed, the heterogeneous expression of psychiatric difficulties in children with AS/HFA may be directly related to environmental factors and this suggests the opportunity of planning differential intervention. For instance, family and daily routines have to be considered as environmental factors that could potentially lead to a different burden of the psychiatric comorbidities.

The challenge of understanding the special needs of a AS/HFA child and the difficulties to build a close relationship with him/her contributes to increase the overall level of stress in the parents, as reported by several studies [77,78]. Parents of children with AS/HFA have been shown to have an impaired sense of well being, and to display a lower quality of life even in comparison with parents of children with other psychiatric and neurological disorders, such as cerebral palsy or mental retardation [79]. Moreover, mothers were found to perceive a higher level of stress than fathers and this maternal impairment was shown to be related to peculiar behavioral characteristics of the child, such as higher hyperactivity and conduct problems [80]. Other studies also reported elevated rates of anxiety-related personality traits among the relatives of AS/HFA participants [81]. Elevated anxiety and stress levels in the parents of AS/HFA children can be considered as an important environmental factor that intersects and acts as trigger in the context of innate genetically determined personality traits - eventually shared with other family members - constituting a genetic family loading for psychiatric conditions. For this reason, investigating the psychiatric family history may inform about the comorbidity risk in the proband as well.

The importance of environmental context in psychiatric symptoms expression has been recently investigated in a sample of youths with ASDs including AS and their siblings, with an evaluation reported independently by parents and teachers. Reports by teachers show a much lower prevalence of psychiatric comorbidity, in particular for affective, anxiety, attention, conduct, oppositional, and somatic problems, in these individuals, as compared to the reports of their parents. These results support the notion that the manifestations of psychopathology in people with AS/HFA may vary depending on the context and that their identification depends on the type of observer, suggesting that informant discrepancies may constitute an additional variable element for the proper identification of comorbidities [70]. In this regard, we have to keep in mind that in the clinical practice there is often a lack of consensus between parent’s and teacher’s reports about the behavioral aspects in children with AS/HFA, thus suggesting caution when making conclusions or interpretations regarding the presence of comorbid psychiatric problems based on a single informant source or just an environmental context, and emphasizing the importance of gathering information from multiple sources and settings, including direct observation by clinicians [70].

The difficulties that a child suffering from ASD experiences in terms of social relationships are likely to be even greater outside of the home environment. The lack of social support, adequate communication and coordination among social service providers often leaves the family alone with the burden of providing any additional support and a more intensive level of care. Indeed, schools are not always ready to cope with the specific needs of AS/HFA and this may drive them to develop feelings of sadness, self-blame and low self-esteem, [82] that may lead to depression, as well as conditions such as hyperactive behaviors or conduct problems. Finally, several studies have reported that negative events such as death, parental discord or frequent changes of own residence may have substantial influence on the child’s mood and functioning, and have been associated with clinical depression [83]. It is likely that AS/HFA participants react to negative life events more severely than individuals from the general population, or, at least, in a different way. This could also be explained by the observation that AS/HFA group seems to be more vulnerable than control individuals to develop mood disorders and depressive symptoms [82], and this vulnerability appears to be correlated to a genetic predisposition.

In conclusion, since the environment seems to considerably influence the expression of psychiatric comorbidities in AS/HFA individuals, more attention should be focused on the interactions between these individuals and their different everyday life environments, in order to provide a better social support and therefore develop coping strategies that could in the end even contribute to a decrease in the incidence of psychiatric disorders in AS/HFA.

## Recommendations and strategies of interventions

As reviewed, several studies investigating the psychiatric comorbidity in individuals with AS/HFA have been conducted. Still, there is uncertainty as to the best way of identifying and conceptualizing psychiatric disorders in AS/HFA.

Although people with AS/HFA can suffer from important comorbidities, according to what Ghazziudin observed, psychiatric disorders go unrecognized in many individuals with AS/HFA [83].

The occurrence of psychiatric disorders in AS/HFA may be the result of a combination of genetic and environmental factor. We believe that an effort should be done to better understand the needs of AS/HFA children in school and family environments, to avoid feelings of low self-esteem and distress, socially inappropriate behaviors, anxiety and other externalizing or internalizing problems. Considering that there is evidence supporting the fact that Asperger syndrome clusters in families and that there is a high prevalence of psychiatric disorders such as depression and schizophrenia in relatives of individuals with AS/HFA [83], the role played by the environment becomes even more relevant on the development and onset of psychiatric conditions in children and adolescents with AS/HFA. Indeed, school and family settings can modulate the behavior of children and adolescent with AS/HFA, thus positively preventing the onset of a psychiatric condition in comorbidity, even in the presence of high genetic predisposition or alternatively precipitating a fragile balance and trigger the development of a psychiatric comorbid condition.

An additional concern regards the suitability of the psychometric tools used in clinical practice to assess comorbid conditions in AS/HFA. Of course, the psychiatric diagnosis of comorbidity relies on the competence of clinicians, but adequate tools such as scales or questionnaires adapted for ASD could be helpful to somehow standardize the recognition of psychiatric symptoms in this clinical sample. So far, most of the studies have used questionnaires, checklists and scales designed for individuals with a normal awareness of their own and other’s mental status. These instruments were not specifically adapted to take into account the neuropsychological characteristics of AS/HFA in terms of deficits in mind reading, impairments in processing and reporting their own feelings, emotions and communication problems. Hence, new psychometric instruments, developed or adapted specifically to AS/HFA, are needed. In the absence of specific tools for ASDs, an alternative strategy could be gathering information from multiple sources and settings, including both direct observation and multiple assessments within- informant and across-informants [70]. We agree with other reports suggesting caution when making conclusions or interpretations regarding the presence of comorbid psychiatric problems based on a single informant source or environmental context, because this may lead to incorrect diagnostic conclusions in particular in ASDs [84,85]. Moreover the current systems of classification do not make it easy to give additional psychiatric diagnoses to persons with AS/HFA. For instance, according to DSM-IV-TR classification, ADHD cannot be diagnosed during the course of a pervasive developmental disorder. Longitudinal studies could allow to understand if individuals with AS may have an increased risk or a constitutive predisposition to develop psychiatric disorders or whether the incidence of the onset is similar to normal population.

### Which is the role-played by medications in the treatment of psychiatric comorbidities?

The proper recognition and diagnosis of psychiatric comorbidities in AS/HFA is also a crucial point for the pharmacologic treatment of these individuals. The correct identification and attribution of overlapping symptoms either to the comorbid disorder or to the AS/HFA itself may contribute to the choice of the most appropriate treatment for each one. However, although in several studies the pharmacologic responses in classical autism have been evaluated, so far only a few studies have investigated the effectiveness of pharmacological treatment in AS/HFA. In addition, the majority of clinical trials conducted on AS/HFA have been focused on drug treatment strategies targeted towards the behavioral symptoms (motor hyperactivity and inattention, interfering stereotypical and repetitive behavior, self-injury, aggression), whereas only few controlled studies included drugs targeted towards the specific psychiatric disorders frequently associated in comorbidity with autistic symptoms.

For these reasons, there is still a huge gap hampering the development of effective treatments for psychiatric disorders associated to AS/HFA. An important practical issue that still needs to be solved is whether the treatments specific for psychiatric disorders that have been found effective in non-ASDs children could eventually have the same effectiveness when the same disorders are present in comorbidity with ASDs. Several examples suggest that, unfortunately, this may not be the case. For instance, in a recent study King and colleagues, considering the suggested similarities between repetitive behavior in ASDs and obsessive-compulsive disorders, investigated the response to citalopram therapy in children with ASD and the results showed no difference in the improvement’s rate of repetitive behavior between the citalopram-treated group and the placebo group [86]. This suggests that the compulsive behaviors observed in ASDs may have a qualitative different nature than those of an obsessive-compulsive disorder. Another report showed the effectiveness of a different medication, (i.e. paroxetine) in improving obsessive-compulsive behavior [87]. However, this study investigated only a single case report and additional studies involving a larger sample are needed to clarify the efficacy of this treatment in the case of comorbid condition with AS. Nevertheless, this report supports the essential role of serotonin in obsessive-compulsive disorders and ASD, in line with the results from other randomized studies showing the effectiveness of paroxetine in the treatment of obsessive-compulsive disorder [87,88] as well as the therapeutic effect of selective serotonin reuptake inhibitors in ASD [89-91]. Besides these preliminary results, we strongly believe that a proper recognition of the psychiatric conditions in comorbidity with ASDs is necessary to allow for a more appropriately targeted treatment. In some cases pharmacological treatment could be effective in improving the psychiatric symptoms typically noticed in AS/HFA that frequently have a negative impact on their quality of life, thus facilitating the adjustment in family, school and community.

## Conclusion

Despite the many challenges that we have outlined in this review, the study of psychiatric comorbid disorders continues to provide an increasingly important contribution to the understanding of the clinical phenomenology of ASDs. In this context, the definition of psychiatric disorders that are often found in association with ASD and of the psychiatric symptoms peculiar to the natural course of ASD itself is of crucial help as it could provide insights regarding the developing pattern of these individuals.

## Competing interests

The authors declare that they have no competing interests.

## Authors’ contributions

LM and LaR overviewed the literature, pulled all the informations together and wrote the manuscript; LiR contributed to theoretical interpretation and final proof reading. Each author read and approved the final version of the manuscript.
